# Fatty Acid Composition and Regulatory Gene Expression in Late-Term Embryos of ACRB and COBB Broilers

**DOI:** 10.3389/fvets.2020.00317

**Published:** 2020-06-25

**Authors:** Shengchen Su, Yidi Wang, Chongxiao Chen, Miyoung Suh, Michael Azain, Woo Kyun Kim

**Affiliations:** ^1^Department of Poultry Science, University of Georgia, Athens, GA, United States; ^2^Department of Food and Human Nutritional Sciences, University of Manitoba, Winnipeg, MB, Canada; ^3^Division of Neurodegenerative Disorders & Canadian Centre for Agri-Food Research in Health and Medicine, St. Boniface Hospital Albrechtsen Research Centre, Winnipeg, MB, Canada; ^4^Department of Animal and Dairy Science, University of Georgia, Athens, GA, United States

**Keywords:** breed, embryo, fatty acids, liver, muscle

## Abstract

Cobb broilers (COBB) have been heavily selected for their production performance in the past several decades, while the Athens Canadian Random Bred (ACRB) chickens, a meat-type breed, have been kept as a non-selected control strain. The purpose of this study was to compare these two lines of chickens at late embryonic development and identify the molecular markers and fatty acid profiles underlining their differences in growth performance due to selection. Fertilized eggs of the ACRB (*n* = 6) and COBB (*n* = 6) were used at 14 and 18 embryonic days. Genes involved in lipogenesis and myogenesis were measured using quantitative real-time reverse transcroption-polymerase chain reaction (RT-PCR), and fatty acid (FA) compositions of egg yolk, muscle, and liver were measured using gas chromatography. COBB had higher egg weight, embryo weight, and breast and fat ratio. The gene expression in the liver showed an interaction between age and breed on *FASN* expression, with the highest level in COBB at E18. ACRB had higher *ApoB* and *MTTP* expression, but lower *SREBP-1* expression compared to COBB. No difference was found in myogenesis gene expression in the muscle between two breeds. For the FA composition, muscle was largely affected by both breed and age. Yolk and liver were affected mainly by breed and age, respectively. Constant interaction effects in docosahexaenoic acid (DHA), indicating the highest level in all the tested tissues of ACRB at E14 and the constant main effects with higher myristic, palmitic, and gondoic, but lower linolenic acid in the liver and yolk of COBB compared to the levels in those of ACRB. Finally, fat accumulation in the liver had no obvious difference between the breeds but was higher when embryo was older. In conclusion, broiler breed affects egg, embryo, and tissue weight, as well as FA composition in initial egg yolk and throughout the embryonic development. The highest docosahexaenoic percentage was observed in ACRB, indicating that genetic selection may result in fatty acid profile changes such as lower DHA content in chicken tissues and eggs.

## Introduction

The Athens Canadian Random Bred (ACRB) is an unselected meat-type chicken control strain that originated from the Ottawa meat Control strain (OC) (1). The OC chicken was developed by the Canada Department of Agriculture's Research Branch using four stains of white plumage birds, which were selected as breeders of ACRB pedigreed eggs in 1955 (2). Because ACRB was maintained with random breeding, it has not been heavily selected for body weight gain as commercial broilers, and the genetics of ACRB has been maintained to eliminate commercial selection. It has been used as a control strain in many poultry research studies to eliminate the environmental effects in breeding programs and to monitor changes in commercial broilers over time (1). The Cobb 500 chicken (COBB), like other commercial strains, was selected on growth rate, feed efficiency, yield, meat quality, and many other aspects. For example, from 1980 to 2010, weights for the Cobb 500 product at 6 weeks of age increased from about 1.13 to 2.50 kg (3). Meanwhile, ACRB maintained a 6-weeks body weight at 594 g (4), indicating that COBB would have dramatic changes in body metabolism compared to ACRB.

A few studies have compared ACRB to other commercial broilers during embryonic development. Christensen et al. (5) demonstrated that during the late embryonic stage (E18–E20) ACRB weight was about 70% that of Arbor Acres (AA) embryos (5). The ACRB also have significant lighter liver and heart weight in this prehatch period, but the relative liver and heart weight to body weight is higher in ACRB. Compared to COBB, ACRB embryos required a longer incubation time. The ACRB chicks hatched with a lighter residual yolk sac as a percentage of chick weight. Other parameters such as egg composition, conductance values, and hatch performance were not different between COBB and ACRB (6). In posthatch development, other studies showed that COBB had larger breast and leg muscles and had a significantly greater fat pad, when compared to ACRB, but had smaller heart and liver as a percentage of body weights (4). The crossbreed of AA × Peterson showed higher expression of c-*fos* and c-*myc* in the liver from E14 to D28 (posthatch) of age. Because these two genes are involved in cell proliferation, the result may explain lower organ weight observed in ACRB (7).

Lipogenesis and fatty acid (FA) oxidation are important metabolic pathways in controlling hepatic triacylglycerol content and body fat accumulation (8, 9). There are about 30% lipids in the egg yolk, which are mainly triacylglycerides, phospholipids, and cholesterol (10). In chickens, more than 90% of the total energy in embryonic development is provided by the yolk lipids (11). However, as the embryo encounters emergence, it starts to decrease lipid metabolism and rely on tissue glycogen (5) or anaerobic catabolism for energy (12). Cori cycling and gluconeogenesis were both estimated higher on E19 compared to E14 (13).

Many research groups have investigated the expression of hepatic lipid metabolism genes between different broiler breeds after hatch (14–16). Their results showed that chicken lines with high fat deposition have higher expression of lipogenesis-related genes at certain posthatch development stages. Hepatic gene expression during embryonic development has been less studied. During embryonic development, chickens with high fat deposition showed higher expression level of fatty acid synthase (*FASN*) (17) and peroxisome proliferator-activated receptor α (*PPAR*α) (8). These results suggested that genetic selection has not only modified feed consumption and body composition after hatch but also affected lipogenesis and lipid metabolism within the egg.

In embryonic stages, myoblasts proliferate and differentiate into myotubes, which form the myofibers. Muscle growth during embryonic development is called hyperplasia, which refers to increase of cell number. Muscle growth in posthatch periods is called hypertrophy, which is increased in size. This is achieved by fusion with satellite cells and increased protein accumulation in myofiber (18). In mammals, the expression of *MYF5* and *MYOD* regulates myoblast development (19). In chickens, the expression of *MYF5* can be detected in somites at as early as Hamburger–Hamilton stage 9, which is about 29–33 h after incubation initiation (20). Other molecules in the Wnt pathway include *PAX3* and *PAX7*, which have similar yet distinct functions in regulating myoblast differentiation and expression of *MYF5* and *MYOD* (21). The hedgehog signaling pathway is acknowledged for its function in inhibiting myogenesis (22).

However, few studies have been conducted to understand the roles of breed and embryonic stage on fatty acid profiles and myogenic and lipogenic genes in key organs between selected and unselected chicken breeds during embryonic development. The purpose of this study was to compare the two breeds, ACRB and COBB, by examining the embryonic growth, gene expression, and fatty acid profiling in the tissues of interest between ACRB and COBB during embryonic development.

## Materials and Methods

### Eggs and Incubation

The ACRB breeders were artificially inseminated with pooled semen 5 days before the incubation. Fertile eggs from the ACRB breeders were collected at the University of Georgia. COBB eggs were collected 2 days before incubation and obtained from Fieldale Farms hatchery (Lavonia, GA). Eggs were stored at 18.4°C and 70% prior to incubation. Thirty eggs from each strain were incubated at 37.5 °C and 53% RH. On E7, unfertile eggs were removed from the incubator and each egg was weighed individually.

### Sample Collection

Yolk samples of both breeds on E0 were collected for lipid profiling. On E14 and E18, six eggs, which were weighed closest to the average weight in each strain, were collected. Egg weight on the sampling day was recorded, and the embryo was removed for further analyses. Embryos were euthanized by cervical dislocation, followed by washing in phosphate-buffered saline (PBS) three times and dried. Embryo weight was recorded, and breast muscle, liver, and abdominal and thigh adipose tissues were removed and weighed. Liver and breast muscle for gene expression were collected, placed in liquid nitrogen, and later stored at −80°C. Breast muscle, liver, fat, and yolk were sampled for lipid profiling and stored at −20°C. Liver specimens collected for histology were fixed in 10% phosphate-buffered formalin (Sigma-Aldrich, St. Louis, MO, USA).

### RNA Extraction

Total RNA was extracted from liver tissue using QIAzol Lysis reagent (Qiagen, Germantown, MD, USA) according to the manufacturer's protocol. RNA quantity and purity were determined using a Nanodrop 1000 spectrophotometer (Thermo Fisher Scientific, Pittsburgh, PA, USA). The cDNA was synthesized from total RNA (2,000 ng) using high-capacity cDNA reverse transcription kits (Thermo Fisher Scientific, Waltham, MA, USA) and was diluted to 6.7 ng/μL for real-time polymerase chain reaction (RT-PCR) analysis.

### Real-Time Quantitative RT-PCR

Hepatic lipid metabolism genes, *ABCA-1* (ATP-binding cassette 1), *ACC* (acetyl coenzyme A carboxylase), *ACLY* (adenosine triphosphate citrate lyase), *ApoB* (apolipoprotein B), *FASN* (fatty acid synthase), malic enzyme, *MTTP* (microsomal triglyceride transfer protein), *PPAR*α (peroxisome proliferator-activated receptor α), *PPAR*γ (peroxisome proliferator-activated receptor γ), and *SREBP-1* (sterol regulatory element-binding protein 1), were analyzed by quantitative real-time PCR (qRT-PCR). Myogenesis genes, *MEF2a* (myocyte enhancer factor 2A), *MEF2c, MYF5* (myogenic factor 5), *MYF6, MYOD* (myoblast determination protein), *MYOG* (myogenin), *PAX3* (paired box protein Pax-3), *PAX7*, and *PTCH* (protein patched homolog), were examined in muscle samples. *GAPDH* was chosen as the reference gene. The forward and reverse primers for the 12 genes are shown in Table 1. All the primers and gene information were designed and obtained from the National Center for Biotechnology Information (NCBI; https://www.ncbi.nlm.nih.gov/). qRT-PCR was performed on an Applied Biosystems StepOnePlus™ (Thermo Fisher Scientific, Waltham, MA, USA) using the following conditions for all genes: 95°C for 10 min followed by 40 cycles of 95°C for 15 s and 60°C for 60 s. The qPCR reaction mixer for each sample included 3.5 μL of iTaq™ Universal SYBR Green Supermix (BioRad, Hercules, CA, USA), 250 nM of each primer, 13.4 ng template, and 4 μL of nuclease-free water (MilliporeSigma, Burlington, MA, USA). Six replicated samples were run in duplicate, and relative gene expression data were analyzed using the 2-ΔΔ*C*_*t*_ method (23). The mean Δ*C*_*t*_ of ACRB at E14 was used to calculate the ΔΔ*C*_*t*_ value.

**Table 1:** Primer sequences.

**Gene**	**Forward primer**	**Reverse primer**	**Product size**	**Genome reference**
*ABCA-1*	CCCAACACCAGGGGAATCTC	CAGTTTCCGCAGTTTTGCCA	135	NM_204145.2
*ACC*	TTGTGGCACAGAAGAGGGAA	GTTGGCACATGGAATGGCAG	161	NM_205505.1
*ACLY*	CAAGACGTGGTGCAGGTAAAG	AGAAGGTTCATCTCGGGAGC	131	NM_001030540.1
*Apo-B*	AACATGGCCCAGTATCAGCC	GTGCCGAGCAGTGATACCAT	137	NM_001044633.1
*FASN*	AGAGGCTTTGAAGCTCGGAC	GGTGCCTGAATACTTGGGCT	127	NM_205155.2
*ME*	GCTTGCCAGCATTACGGTTT	TGTCCCCGGTCATGGATAGT	71	NM_204303.1
*MTTP*	CGTCAAGAACCGGATAGCCA	CTGTGAAAACTGCACCGTGG	133	NM_001109784.2
*PPAR-α*	CACTTTTTGTCGCTGCCATCA	GCCGGAGGTCAGCCATTTTT	177	NM_001001464.1
*PPAR-γ*	TGAATGTCGTGTGTGTGGGG	GCATTCGCCCAAACCTGATG	230	NM_001001460.1
*SREBP-1*	TTCTCAGGGCTGTTCGATGC	AACACATTGCCGGTAGGGGG	119	NM_204126.2
*MEF2a*	CTGGCAATGCTGGTGGAATG	ATTCATCCTCCTCCGTCAGT	279	NM_204864.3
*MEF2c*	TAGGTCACAGCCCTGAGTCT	ATGGGCCAGTGGCAAAAGAT	203	XM_004949410.3
*MTF5*	GAGGAACGCCATCAGGTACATC	ACATCGGAGCAGCTGGAGCT	126	NM_001030363.1
*MYF6*	AGGACAAAATGCAGGAGGTG	TCGTCGGAGGAAATGCTGTC	229	NM_001030746.1
*MYOD*	GACAGCAGCTACTACACGGAATCA	GGAAATCCTCTCCACAATGCTT	102	NM_204214.2
*MYOG*	AGCAGCCTCAACCAGCAGGA	TCTGCCTGGTCATCGCTCAG	179	NM_204184.1
*PAX3*	ACTACCCTGACATTTATACTCG	TGCCTGCTTCCTCCATCTAG	110	NM_204269.1
*PAX7*	ATGAATCCTGTTAGCAATGGC	GGAGAGATGGAGAAGTCAGC	101	NM_205065.1
*PTCH*	GGCGTTCGCGGTGGGACTAC	GGTGCTGCCGGAGTGCTTCT	106	NM_204960.2
*GAPDH*	GCTAAGGCTGTGGGGAAAGT	TCAGCAGCAGCCTTCACTAC	161	NM_204305.1

*ABCA-1, ATP-binding cassette 1; ACC, acetyl coenzyme A carboxylase; ACLY, adenosine triphosphate citrate lyase; ApoB, apolipoprotein B; FASN, fatty acid synthase; GAPDH, glyceraldehyde 3-phosphate dehydrogenase; MEF2a, myocyte enhancer factor 2A; MEF2c, myocyte enhancer factor 2C; MYF5, myogenic factor 5; MYF6, myogenic factor 6; MYOD, myoblast determination protein; MYOG, myogenin; MTTP, microsomal triglyceride transfer protein; PAX3, paired box protein Pax-3; PAX7, paired box protein Pax-7; PPARα, peroxisome proliferator-activated receptor α; PPARγ, peroxisome proliferator-activated receptor γ; PTCH, protein patched homolog; SREBP-1, sterol regulatory element-binding protein 1*.

### Fatty Acid Composition

To measure fatty acid composition, a direct saponification and methylation method was used for egg yolk, muscle, and liver with a slight modification of method developed by (24). In brief, saponification was carried out for all samples by adding 0.5 M methanolic KOH at 110 °C for 1 h, followed by methylation. The separation of fatty acid methyl esters was carried out on a SGE BPX-70 column (10 m × 0.10 mm diameter and 0.2 m film thickness)using a Bruker 450 gas chromatography instrument coupled to a flame ionization detector (Bruker, CompassXport 3.0, Billerica, MA, USA). The detailed run conditions have been published elsewhere (25).

### Histology

Liver tissues collected on E14 and E18 were fixed in 10% phosphate-buffered formalin and were sent to the Poultry Diagnostic and Research Center at the University of Georgia for processing. In brief, samples were embedded in paraffin, cut into sections at 4.0 μm thickness, and stained with standard hematoxylin and eosin solution. Slides were examined with an Olympus IX71 inverted research microscope, using bright-field observation.

### Statistical Analysis

Gene expression results and egg, embryo, and tissue weights were analyzed by two-way ANOVA using JMP^®^ Statistical Discovery Software from SAS (SAS Institute, Cary, NC, USA). The model contains main effects of breed and embryonic day, and interaction between the two variables. Individual means were further analyzed using Tukey's HSD test (*P* < 0.05). FA profiling data were analyzed by *t*-test for egg yolk at the baseline and two-way ANOVA for other tissues.

## Results

### Egg, Embryo, and Tissue Weight

Egg weight, embryo weight, embryo: egg weight ratio, and tissue: embryo weight ratio are shown in Table 2. There were no significant interactions in any parameters. COBB eggs were 14.87 g (31%) and 14.26 g (30%) heavier than ACRB egg on E14 and E18 (*P* < 0.0001), respectively. COBB embryos were 5.97 g (56%) and 6.91 g (31%) heavier than ACRB on E14 and E18 (*P* < 0.0001), respectively. Embryo weight relative to egg weight was higher on E18 than E14 without breed difference (*P* < 0.0001). Relative breast muscle was higher on E14 when compared to E18 (*P* < 0.0001), and COBB had heavier relative breast muscle than ACRB on E14 (*P* = 0.0016); however, no breed difference was observed on E18. Relative liver weight was significantly higher on E18, whereas no breed difference was observed on either day. Relative fat weight was higher on E18 than E14 (*P* = 0.03), and COBB embryos had higher fat tissue weight than ACRB on E18 (*P* = 0.0002).

**Table 2:** Egg, embryo, and relative embryo and tissue weight on embryonic days 14 and 18.

	**E14**		**E18**		***P*****-Value**		
	**ACRB**	**COBB**	**ACRB**	**COBB**	**Breed**	**Day**	Breed*Day
Egg WT (g)	47.77 ± 0.89^b^	62.64 ± 0.93^a^	47.01 ± 0.84^b^	61.27 ± 0.83^a^	<0.0001	NS	NS
Embryo WT (g)	10.72 ± 0.22^d^	16.69 ± 0.31^c^	22.32 ± 1.32^b^	29.23 ± 1.14^a^	<0.0001	<0.0001	NS
% Embryo^1^	22.48 ± 0.76^b^	26.64 ± 0.33^b^	47.48 ± 2.68^a^	47.71 ± 1.79^a^	NS	<0.0001	NS
% Breast^2^	2.26 ± 0.12^b^	2.71 ± 0.11^a^	1.04 ± 0.07^c^	1.28 ± 0.11^c^	0.0016	<0.0001	NS
% Liver^2^	1.83 ± 0.08^a^^,^^b^	1.64 ± 0.1^b^	1.92 ± 0.11^a^^,^^b^	2.11 ± 0.17^a^	NS	0.02	NS
% Fat^2^	0.84 ± 0.08^b^	1.08 ± 0.1^b^	0.89 ± 0.07^b^	1.44 ± 0.13^a^	0.0002	0.03	NS

*Values within a row having different superscript letters are statistically different by Tukey's HSD test (P < 0.05). ACRB, Athens Canadian Random Bred meat-type chicken. NS, not significant*.

a Relative embryo weight (%) = (embryo weight/egg weight)^*^100.

b Relative tissue weight (%) = (tissue weight/embryo weight)^*^100.

### Gene Expression in Liver and Muscle

**Figure 1:**
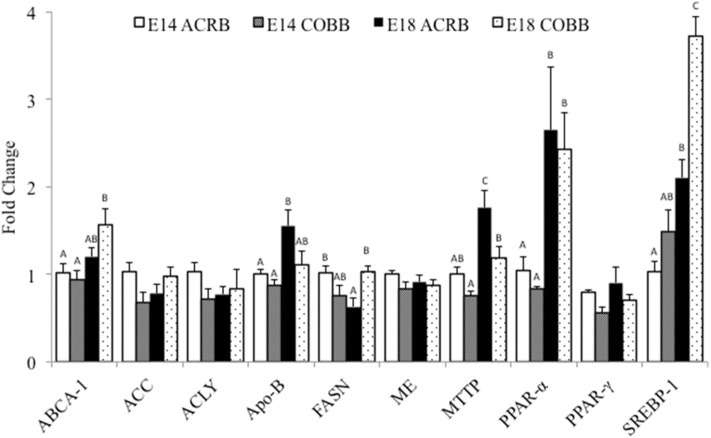
Gene expression in the liver of ACRB and Cobb broilers on E14 and E18. Different letters with each gene indicate statistical differences in expression level (*P* < 0.05). The bar graph shows the means±SE. *ABCA-1*, ATP-binding cassette 1; *ACC*, acetyl coenzyme A carboxylase; *ACLY*, adenosine triphosphate citrate lyase; *ApoB*, apolipoprotein B; *FASN*, fatty acid synthase; *MTTP*, microsomal triglyceride transfer protein; *PPAR*α, peroxisome proliferator-activated receptor α; *PPAR*γ, peroxisome proliferator-activated receptor γ; *SREBP-1*, sterol regulatory element-binding protein 1.

Comparison of hepatic lipogenesis and lipid metabolism related gene expression between ACRB and COBB is shown in Figure 1. The main effect of embryonic day was observed in *ABCA-1, ApoB, MTTP, PPAR-*α, and *SREBP*; the expression of these genes was significantly higher on E18 when compared to E14. The main effect of breed was shown in *ApoB* and MTTP; the gene expression was significantly higher in ACRB. The main effect of breed was also shown in *SREBP-1* expression, which was significantly higher in COBB. The only interaction of embryonic age and breed was observed in *FASN* expression.

**Figure 2:**
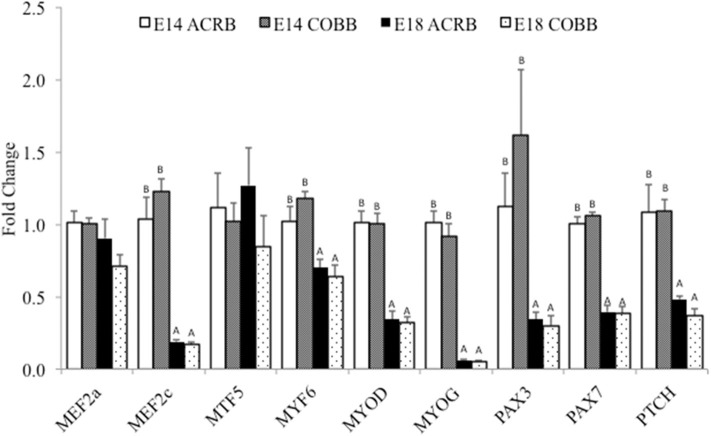
Gene expression in the muscle of ACRB and Cobb broilers on E14 and E18. Different letters with each gene indicate statistical differences in expression level (*P* < 0.05). The bar graph shows the means±SE. *MEF2a*, myocyte enhancer factor 2A; *MEF2c*, myocyte enhancer factor 2C; *MYF5*, myogenic factor 5; *MYF6*, myogenic factor 6; *MYOD*, myoblast determination protein; *MYOG*, myogenin; *PAX3*, paired box protein Pax-3; *PAX7*, paired box protein Pax-7; *PTCH*, protein patched homolog.

A comparison of key myogenic genes between ACRB and COBB is shown in Figure 2. The main effect of embryonic day was observed in *MEF2c, MYF6, MYOD, MYOG, PAX3, PAX7*, and *PTCH* expression, which indicates significantly higher expression levels of these genes on E14 compared to E18. No effect of breed or interaction was detected in myogenesis gene expression.

### Fatty Acid Composition Analysis

FA composition (mol %, wt/wt) of egg yolk prior to incubation is shown in Table 3. Egg yolk at E0 was abundant in oleic acid followed by palmitic acid. linoleic acid, an essential n-6 FA, was the dominant among other polyunsaturated fatty acids (PUFAs). Compared to ACRB at this age, COBB had lower level of oleic acids (33.63 vs. 42.99%, *P* < 0.001) and total monounsaturated FAs (37.01 vs. 46.37%, *P* < 0.001), whereas they had higher essential FAs linoleic (22.36 vs. 15.56%, *P* < 0.01), γ-linolenic (0.31 vs. 0.18, *P* < 0.01), and α-linolenic acids (1.18 vs. 0.60%, *P* < 0.001), leading to higher total n-6 (26.62% vs. 19.18, *P* < 0.01) and n-3 FA (2.78 vs. 2.07%, *P* < 0.01).

**Table 3:** Fatty acid composition of egg yolk from ACRB and Cobb broilers at E0.

**Fatty acid (mol %)**		**ACRB (*****n*** **=** **6)**		**Cobb (*****n*** **=** **6)**	
Myristic	14:0	0.39 ±	0.02	0.42 ±	0.12
Palmitic	16:0	22.73 ±	2.37	23.76 ±	1.00
Palmitoleic	16:1n-7	2.93 ±	0.26	2.85 ±	0.49
Margaric	17:0	0.11 ±	0.06	0.20 ±	0.11
Stearic	18:0	9.10 ±	0.85	9.01 ±	0.38
Oleic	18:1n-9	42.99 ±	2.62	33.63 ±	1.48^***^
Linoleic	18:2n-6	15.56 ±	1.93	22.36 ±	2.58^**^
γ-Linolenic	18:3n-6	0.18 ±	0.05	0.31 ±	0.03^**^
α-Linolenic	18:3n-3	0.60 ±	0.08	1.18 ±	0.17^***^
Arachidic	20:0	0.01 ±	0.00	0.02 ±	0.03
Gondoic	20:1n-9	0.29 ±	0.03	0.32 ±	0.01
Eicosadienoic	20:2n-6	0.01 ±	0.00	0.01 ±	0.01
Dihomo-γ-linolenic	20:3n-6	0.14 ±	0.01	0.27 ±	0.02^***^
Arachidonic	20:4n-6	2.21 ±	0.12	2.45 ±	0.30
Eicosapentaenoic	20:5n-3	0.03 ±	0.00	0.04 ±	0.01^*^
Docosahexaenoic	22:6n-3	1.25 ±	0.12	1.25 ±	0.11
Total SFA		32.38 ±	2.48	33.58 ±	1.18
Total mono		46.37 ±	2.75	37.01 ±	1.91^***^
Total n-6 FA		19.18 ±	1.89	26.62 ±	2.72^**^
Total n-3 FA		2.07 ±	0.17	2.78 ±	0.26^**^

*Values are means ± SD. Significant differences between the strains were identified by t-test*;

* *P < 0.05*;

** *P < 0.01*;

*** *P < 0.001*.

*ACRB, Athens Canadian Random Bred meat-type chicken; mono, monounsaturated fatty acids; SFA, saturated fatty acid*.

FA composition of egg yolk on E14 and E18 is shown in Table 4. While the major FAs are the same as at E0, the age differences were not identified in all of the FAs analyzed except docosahexaenoic acid (DHA, C22:6n-3, *P* = 0.027), a major n-3 PUFA showing significant breed and age interaction effect (*P* = 0.025); ACRB on E14 had significantly higher DHA but lower at E18 compared to COBB on the same age. Arachidonic acid level was decreased with age. Compared to ACRB, COBB had higher myristic, palmitic, margaric, γ-linolenic (18:3n-6), gondoic acids, and total saturated fatty acids (SFAs), but had lower palmitoleic, linoleic, α-linolenic (18:3n-3) and total n-6 FA.

**Table 4:** Fatty acid composition of the yolk from ACRB and Cobb broiler embryos at E14 and E18.

		**E14**				**E18**				***P*****-Value**		
**Fatty acid (mol %)**		**ACRB (*****n*** **=** **6)**		**Cobb (*****n*** **=** **6)**		**ACRB (*****n*** **=** **6)**		**Cobb (*****n*** **=** **6)**		**Breed**	**Age**	Breed*Age
Myristic	14:0	0.30 ±	0.01^b^	0.45 ±	0.02^a^	0.34 ±	0.02^b^	0.45 ±	0.01^a^	<0.0001	NS	NS
Palmitic	16:0	24.22 ±	0.42^b^	26.71 ±	0.52^a^	24.87 ±	0.48^b^	26.66 ±	0.54^a^	0.0001	NS	NS
Palmitoleic	16:1n-7	2.99 ±	0.18^b^	3.91 ±	0.34^a, b^	3.13 ±	0.23^a, b^	3.95 ±	0.30^a^	0.002	NS	NS
Margaric	17:0	0.18 ±	0.01	0.19 ±	0.01	0.18 ±	0.01	0.24 ±	0.03	0.04	NS	NS
Stearic	18:0	8.55 ±	0.35	9.11 ±	0.22	8.16 ±	0.40	8.80 ±	0.28	NS	NS	NS
Oleic	18:1n-9	40.27 ±	1.31	39.10 ±	0.86	40.52 ±	1.13	38.89 ±	0.39	NS	NS	NS
Linoleic	18:2n-6	17.17 ±	1.43^a, b^	14.40 ±	0.34^b^	17.67 ±	0.77^a^	14.30 ±	0.52^b^	0.001	NS	NS
γ-Linolenic	18:3n-6	0.04 ±	0.01	0.06 ±	0.01	0.04 ±	0.00	0.06 ±	0.01	0.01	NS	NS
α-Linolenic	18:3n-3	0.64 ±	0.09^a^	0.38 ±	0.02^b^	0.67 ±	0.04^a^	0.37 ±	0.02^b^	<0.0001	NS	NS
Arachidic	20:0	0.03 ±	0.00	0.04 ±	0.00	0.04 ±	0.01	0.03 ±	0.00	NS	NS	NS
Gondoic	20:1n-9	0.22 ±	0.02^c^	0.29 ±	0.01^a, b^	0.23 ±	0.03^b, c^	0.30 ±	0.01^a^	0.0003	NS	NS
Eicosadienoic	20:2n-6	0.17 ±	0.03	0.17 ±	0.01	0.16 ±	0.03	0.18 ±	0.02	NS	NS	NS
Arachidonic	20:4n-6	2.16 ±	0.11	2.16 ±	0.09	1.89 ±	0.16	1.95 ±	0.10	NS	0.04	NS
Eicosapentaenoic	20:5n-3	0.87 ±	0.14	0.59 ±	0.12	0.54 ±	0.20	1.51 ±	0.68	NS	NS	NS
Docosahexaenoic	22:6n-3	0.81 ±	0.06^a^	0.41 ±	0.03^b^	0.37 ±	0.07^b, c^	0.20 ±	0.03^c^	<0.0001	<0.0001	0.027
Total SFA		33.28 ±	0.67^b^	36.49 ±	0.40^a^	33.60 ±	0.70^b^	36.17 ±	0.57^a^	<0.0001	NS	NS
Total mono		43.67 ±	1.38	43.56 ±	0.61	44.08 ±	1.33	43.47 ±	0.49	NS	NS	NS
Total n-6 FAs		19.54 ±	1.42^a, b^	16.80 ±	0.33^a, b^	19.75 ±	0.84^a^	16.49 ±	0.51^b^	0.001	NS	NS
Total n-3 FAs		2.31 ±	0.16	1.38 ±	1.03	1.57 ±	0.21	2.08 ±	0.67	NS	NS	0.05

*Values are means ± SD. Significant effects were identified by two-way analysis of variance procedures for breed and age. Values within a row having different superscript letters are different (P < 0.05). ACRB, Athens Canadian Random Bred meat-type chicken; FAs, fatty acids; mono, monounsaturated fatty acids; SFA, saturated fatty acid. NS, not significant*.

In the liver (Table 5), unlike egg yolk, both n-6 and n-3 PUFA became the major fatty acids, especially with arachidonic acid and DHA. In terms of age differences, the most pronounced difference was identified in oleic acid, which increased to a level 2.4–2.5 times higher in E18 in comparison to E14 in both breeds (*P* < 0.0001). At E18, both n-6 and n-3 essential FAs, linoleic, and α-linolenic acids were also significantly (*P* < 0.0001) increased in both breeds, while saturated, arachidonic, acid and DHA levels decreased. In terms of breed difference, compared to ACRB, COBB had significantly higher palmitic, plamitoleic, godonic and eicosadienoic acids but lower stearic acid, α-linolenic acid, and DHA.

**Table 5:** Fatty acid composition of the liver from ACRB and Cobb broiler embryos at E14 and E18.

		**E14**				**E18**				***P*** **value**		
**Fatty acid (mol %)**		**ACRB (*****n*** **=** **6)**		**Cobb (*****n*** **=** **6)**		**ACRB (*****n*** **=** **6)**		**Cobb (*****n*** **=** **6)**		**Breed**	**Age**	Breed*Age
Myristic	14:0	0.14 ±	0.06^b,c^	0.19 ±	0.05^a,b^	0.08 ±	0.04^c^	0.23 ±	0.08^a^	0.001	NS	NS
Palmitic	16:0	16.75 ±	1.31^b^	18.40 ±	0.66^a^	9.93 ±	1.64^c^	10.85 ±	0.83^c^	0.01	<0.0001	NS
Palmitoleic	16:1n-7	0.14 ±	0.04^c^	0.37 ±	0.13^b^	0.36 ±	0.08^b^	1.44 ±	0.22^a^	<0.0001	<0.0001	<0.0001
Margaric	17:0	0.19 ±	0.21	0.18 ±	0.17	0.27 ±	0.12	0.11 ±	0.12	NS	NS	NS
Stearic	18:0	17.73 ±	1.08^a^	16.30 ±	0.76^b^	11.48 ±	1.76^c^	10.72 ±	0.79^c^	0.03	<0.0001	NS
Oleic	18:1 n-9	18.66 ±	3.09^b^	18.07 ±	1.27^b^	43.99 ±	5.93^a^	45.73 ±	3.38^a^	NS	<0.0001	NS
Linoleic	18:2n-6	9.20 ±	1.71^b^	9.51 ±	1.21^b^	13.25 ±	1.85^a^	12.01 ±	0.61^a^	NS	<0.0001	NS
γ-Linolenic	18:3n-6	0.03 ±	0.02^b^	0.04 ±	0.02^a,b^	0.05 ±	0.03^a,b^	0.08 ±	0.05^a^	NS	0.05	NS
α-Linolenic	18:3n-3	0.14 ±	0.06^c^	0.06 ±	0.03^d^	0.29 ±	0.06^a^	0.22 ±	0.04^b^	0.001	<0.0001	NS
Arachidic	20:0	0.09 ±	0.10^a,b^	0.15 ±	0.05^a^	0.04 ±	0.03^b^	0.04 ±	0.03^b^	NS	0.005	NS
Gondoic	20:1n-9	0.23 ±	0.05^b^	0.32 ±	0.02^a^	0.21 ±	0.03^b^	0.33 ±	0.04^a^	<0.0001	NS	NS
Eicosadienoic	20:2n-6	0.01 ±	0.01^b^	0.03 ±	0.01^b^	0.03 ±	0.03^b^	0.07 ±	0.01^a^	0.003	0.01	NS
Arachidonic	20:4n-6	20.22 ±	1.00^a^	20.17 ±	1.11^a^	10.66 ±	1.38^b^	9.55 ±	1.12^b^	NS	<0.0001	NS
Eicosapentaenoic	20:5n-3	0.17 ±	0.09	0.12 ±	0.07	0.13 ±	0.03	0.12 ±	0.01	NS	NS	NS
Docosahexaenoic	22:6n-3	11.47 ±	1.21^a^	7.42 ±	1.06^b^	6.14 ±	0.66^c^	4.00 ±	0.77^d^	<0.0001	<0.0001	0.025
Total SFA		35.32 ±	2.22^a^	35.77 ±	1.17^a^	22.02 ±	3.01^b^	22.18 ±	1.44^b^	NS	<0.0001	NS
Total mono		19.75 ±	3.10^b^	19.15 ±	1.32^b^	45.07 ±	5.94^a^	47.83 ±	3.47^a^	NS	<0.0001	NS
Total n-6 FAs		32.61 ±	1.42^b^	36.70 ±	1.59^a^	25.95 ±	2.86^c^	25.31 ±	1.61^c^	0.05	<0.0001	0.008
Total n-3 FAs		12.32 ±	1.21^a^	8.38 ±	1.08^b^	6.95 ±	0.68^c^	4.69 ±	0.81^d^	<0.0001	<0.0001	0.05

*Values are means ± SD. Significant effects were identified by two-way analysis of variance procedures for breed and age. Values within a row having different superscript letters are different (P < 0.05). ACRB, Athens Canadian Random Bred meat-type chicken; FAs, fatty acids; mono, monounsaturated fatty acids; SFA, saturated fatty acid. NS, not significant*.

In the muscle (Table 6), palmitoleic, stearic, oleic, linoleic, α-linolenic (18:3n-3), gondoic, arachidonic, and eicosapentaenoic acids, DHA, total SFA, total mono, and total n-3 FAs showed breed and age interaction. Similar to the result of the liver, ACRB on E14 had significantly higher DHA (*P* = 0.004) and total n-3 FA (*P* = 0.003) compared to the other groups. α-linolenic (18:3n-3), gondoic, and eicosadienoic acids and DHA total n-3 FAs and total n-6 FAs showed a breed difference. Myristic, palmitic, linoleic, gondoic, and total n-6 FA showed an age difference.

**Table 6:** Fatty acid composition of the muscle from ACRB and Cobb broiler embryos at E14 and E18.

		**E14**				**E18**				***P*****-value**		
**Fatty acid (mol %)**		**ACRB (*****n*** **=** **6)**		**Cobb (*****n*** **=** **6)**		**ACRB (*****n*** **=** **5)**		**Cobb (*****n*** **=** **6)**		**Breed**	**Age**	Breed*Age
Myristic	14:0	0.49 ±	0.11^a^	0.51 ±	0.16^a^	0.45 ±	0.18^a^	0.23 ±	0.12^b^	NS	0.02	NS
Palmitic	16:0	24.22 ±	0.29^a,b^	25.54 ±	1.03^a^	23.45 ±	1.66^b^	23.38 ±	1.00^b^	NS	0.005	NS
Palmitoleic	16:1n-7	0.39 ±	0.11^a,b^	0.83 ±	0.46^a^	0.67 ±	0.48^a,b^	0.35 ±	0.21^b^	NS	NS	0.02
Margaric	17:0	0.03 ±	0.02	0.18 ±	0.24	0.17 ±	0.25	0.02 ±	0.01	NS	NS	NS
Stearic	18:0	15.58 ±	0.68^a,b^	13.43 ±	2.07^b^	13.92 ±	2.15^b^	16.91 ±	1.42^a^	NS	NS	0.002
Oleic	18:1n-9	20.85 ±	1.62^b^	23.22 ±	4.66^a,b^	25.99 ±	3.59^a^	20.05 ±	3.34^b^	NS	NS	0.01
Linoleic	18:2n-6	8.91 ±	1.86^b^	9.55 ±	2.34^b^	15.22 ±	3.42^a^	10.45 ±	0.88^b^	NS	0.002	0.01
γ-Linolenic	18:3n-6	0.09 ±	0.07	0.11 ±	0.07	0.09 ±	0.06	0.03 ±	0.01	NS	NS	NS
α-Linolenic	18:3n-3	0.13 ±	0.18^b^	0.19 ±	0.18^b^	0.51 ±	0.26^a^	0.03 ±	0.02^b^	0.02	NS	0.003
Arachidic	20:0	0.38 ±	0.16	0.24 ±	0.21	0.17 ±	0.29	0.39 ±	0.03	NS	NS	NS
Gondoic	20:1n-9	0.38 ±	0.03^b^	0.44 ±	0.02^a^	0.29 ±	0.04^c^	0.40 ±	0.02^a,b^	<0.0001	<0.0001	0.03
Eicosadienoic	20:2n-6	0.02 ±	0.01^ab^	0.03 ±	0.01^a^	0.01 ±	0.02^b^	0.03 ±	0.01^a^	0.02	NS	NS
Arachidonic	20:4n-6	12.42 ±	0.97^a^	11.20 ±	2.90^a,b^	8.59 ±	3.10^b^	12.67 ±	1.56^a^	NS	NS	0.01
Eicosapentaenoic	20:5n-3	0.32 ±	0.03^a^	0.22 ±	0.07^b^	0.22 ±	0.09^b^	0.28 ±	0.05^a,b^	NS	NS	0.01
Docosahexaenoic	22:6n-3	8.76 ±	0.83^a^	5.45 ±	1.29^b^	5.25 ±	1.60^b^	5.19 ±	0.72^b^	0.003	0.001	0.004
Total SFA		41.90 ±	1.27^a^	40.88 ±	1.57^a^	38.90 ±	2.10^b^	41.80 ±	0.53^a^	NS	NS	0.006
Total mono		22.75 ±	1.84^b^	25.22 ±	4.88^a,b^	27.60 ±	3.85^a^	21.55 ±	3.46^b^	NS	NS	0.01
Total n-6 FAs		25.37 ±	1.80^b^	27.36 ±	2.30^b^	26.92 ±	1.62^b^	30.13 ±	2.51^a^	0.007	0.02	NS
Total n-3 FAs		9.98 ±	0.87^a^	6.55 ±	1.29^b^	6.57 ±	1.58^b^	6.52 ±	0.70^b^	0.002	0.003	0.003

*Values are means ± SD. Significant effects were identified by two-way analysis of variance procedures for breed and age. Values within a row having different superscript letters are different (P < 0.05). ACRB, Athens Canadian Random Bred meat-type chicken; FAs, fatty acids; mono, monounsaturated fatty acids; SFA, saturated fatty acid. NS, not significant*.

### Histology

**Figure 3:**
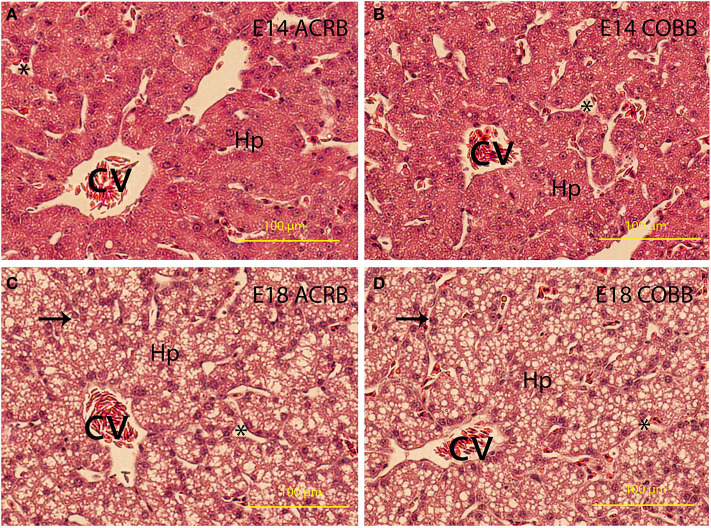
Liver development and lipid deposition. The embryonic day and breed information are indicated in the upper right corner in each picture. Arrows indicate lipid deposit in hepatocytes. CV, central vein; Hp, hepatocytes. ^*^Indicates sinusoidal capillaries. Bar is 200 μm.

Little differences in lipid deposition were observed between the ACRB (Figures 3A,C) and COBB (Figures 3B,D) chicken at the same age of embryonic development. However, lipid deposition on E18 was considerably higher than E14 regardless of the breeds.

## Discussion

Egg characteristics and hatch performance of ACRB and COBB were first reported by Collins et al. (4). In their study, egg composition, conductance values, incubation duration, hatch performance, and yolk utilization were measured (6). The results showed that COBB had higher egg weight and relative albumen, but lower initial yolk weight. Compared to ACRB, COBB also has higher final relative yolk weight, indicating that ACRB chickens utilized more yolk during embryonic development. However, no information on embryo weight or organ/tissue weight was indicated in their comparison of ACRB and COBB broilers. From E14 to E18, the relative embryo weight and relative liver weight increased; this result is also supported by Pulikanti et al. (26), who evaluated the effect of hen age on embryo development from E15 to E19 (26). In addition, they also observed a decrease in relative yolk weight and increase in pipping muscle weight as incubation proceeded. However, no breed difference was observed on either sampling day. In our current study, parameters associated with production, such as egg weight, embryo weight, breast muscle and fat, were high in COBB on at least one time point. These results indicated that selection of growth does affect embryonic development of different breeds of chickens.

**Figure 4:**
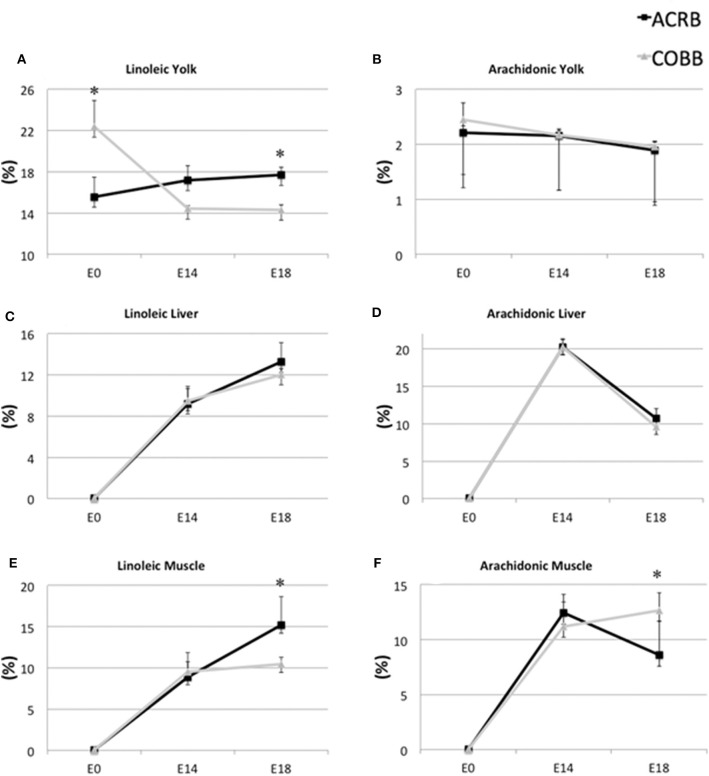
Dynamic distribution of linoleic and arachidonic acid in chicken embryo. Black squares represent ACRB; gray triangles represent Cobb chicken. The *y*-axis is the percentage of the indicated fatty acid in total fatty acid of the designated tissue. ^*^Indicates statistical difference of the same age (*P* < 0.05). The graph shows the means ± SE. **(A)** linoleic acid in the yolk; **(B)** arachidonic acid in the yolk; **(C)** linoleic acid in the liver; **(D)** arachidonic acid in the liver; **(E)** linoleic acid in the muscle; **(F)** arachidonic acid in the muscle.

The yolk supports the embryonic development of avian species throughout the entire incubation phase. However, rapid absorption of yolk nutrients takes place after the yolk sac has surrounded the yolk on E11 (11, 27). Lipids from yolk are absorbed by endocytosis of lipoprotein, as no receptors for free FAs have been found so far (27). Some FAs are synthesized at the yolk sac membrane from other FAs. For example, linoleic acid is converted to arachidonic acid by Δ6-desaturase (28). ACRB had higher linoleic acid in the yolk (Figure 4A) and breast muscle (Figure 4E) on E18, but no different in the liver (Figure 4C) on E18, but COBB showed higher arachidonic acid level in muscle (Figure 4F), despite there being no difference in arachidonic acid level in the yolk (Figure 4B) and muscle (Figure 4D) for the two breeds. This may be because COBB has higher Δ6-desaturase activity. This result is supported by Cherian and Sim (29), who found that chicks hatched from eggs enriched with n-3 fatty acid had lower Δ6-desaturase activity (29).

FA composition differences in the liver were largely affected by embryonic age (Table 6). Fatty acid composition in the breast muscle showed the most breed and age interaction effects (Table 4). The absolute embryonic breast muscle weight is greater in COBB when compared to ACRB (data not shown), and at 6 weeks of age, the weight of breast muscle in COBB is twice that of ACRB (4). Hence, as the embryo develops, the COBB breed grows more muscle and deposits more saturated FAs and n-6 FAs in the muscle. Because high dietary n-6/n-3 ratio may affect obesity (30), these results may explain the heavier fat tissue in COBB chicken in embryonic and posthatch development.

Hepatic triglyceride homeostasis is maintained by regulating FA uptake, *de novo* lipogenesis, and FA export and/or oxidation (31). Our results demonstrated that ACRB expressed higher levels of *ApoB* and *MTTP* on E18 when compared to COBB. *MTTP* plays an important role in synthesis, translocation, and secretion of *ApoB*-containing lipoproteins in the cell (32). This result may be partially explained by the fact that ACRB utilizes more yolk than COBB, as described above. Some evidence showed that *ApoB* also highly expressed in the yolk sac during embryonic development (32). However, we only investigated gene expression in the liver in this study. COBB has increased expression of *ABCA-1* on E18 compared to ACRB (Figure 2). *ABCA-1* interacts with *ApoA-1* and controls efflux of cholesterol (33). Since *ApoB* expression in this study was higher in ACRB, detection of *ApoA-1* should be included in future studies to help to understand the expression pattern of *ABCA-1* during embryonic development.

ACC and ACLY are both involved in FA/lipid synthesis. ACLY catalyzes the reaction that converts citrate to acetyl-CoA, and ACC carboxylates acetyl-CoA to malonyl-CoA, which is the rate-limiting step in FA synthesis. Both acetyl-CoA and malonyl-CoA are substrates of *FASN*, and the complex they form gives rise to palmitate as the precursor for many other FAs (34). ME catalyzes the reaction that generates NADPH, which is used by *FASN* in synthesizing palmitate (35). In the current study, higher expression of *FASN* may indicate that COBB embryos have potentially higher FA synthesis on E18.

In mammals, there are three *SREBP* isoforms, designated as *SREBP-1a, SREBP-1c*, and *SREBP-2*. *SREBP-2* mainly regulates the synthesis and metabolism of cholesterol, while *SREBP-1c* preferentially regulates fatty acid synthesis (36, 37). The function of *SREBP-1c* is not as restricted as that of the other two, which is also involved in adipocyte differentiation and lipogenesis in the cell (38, 39).

Chickens only have one *SREBP-1* isoform, and it is highly homologous to the *SREBP-1a* in mammals (13). In chick embryo hepatocytes, *SREBP-1* enhances the expression of *ACC* by interacting with the nuclear thyroid hormone receptor (40). In the present study, *SREBP-1* showed higher expression in COBB embryos on E14 and E18, which may correspond to the elevated *FASN* on E18.

In breast muscle, no breed difference was observed, and gene expression was affected mainly by the age of embryo, which is at a higher expression level at E14 than at E18. However, these tested genes have various functions in muscle development. *PAX3* and *PAX7* promote the proliferation of either satellites cells or C2C12 myoblasts (21); *MYF5* and *MYOD1* are associated with myoblast proliferation, and *MYOG* regulates myoblast differentiation (41). *MYF6* promotes the expression of muscle proteins and other related muscle-specific genes through trans-activation (42). However, *PTCH-1* represses the function of *MYOD* in myogenesis (22, 43). We did not expect that genes with opposite function in regulating myogenesis would have the same expression pattern. One of the reasons may be that as the embryo developed toward hatching, many of the genes involved in myoblast proliferation and differentiation are downregulated, and muscle growth is shifted to hypertrophy. Furthermore, in E19 chicken, factors promote proliferation, and suppressors for differentiation are presented in the embryo. In contrast, an E10 embryo only has factors that promote mitogenesis (44). These findings may be supporting the results of declined expression of many proliferation-related genes on E18 when compared to E14. Future studies should examine the expression of genes in protein synthesis and satellite cell specific myogenesis factors.

In conclusion, broiler breed affects egg, embryo, and tissue weight, as well as fatty acid composition in initial egg yolk and throughout embryonic development. The COBB chicken was selected for production traits; thus, it has higher egg, embryo, breast muscle, and fat weight. Both breed and age affect most of the gene expression changes in liver lipid metabolism. The changes in muscle gene expression are mainly affected by age. In the initial egg yolk, COBB chicken has lower monounsaturated FA levels but more n-6 and n-3 FAs. During development, ACRB has higher n-6 FA content in the yolk compared to COBB. Unlike in yolk, the ACRB liver contains a higher n-3 FA content than COBB. An interesting constant interaction effect was found in DHA level, which was the highest in all the tested tissues of ACRB at E14. More research needs to be conducted to define the role of fatty acids in embryo development.

## Data Availability Statement

The datasets generated for this study are available on request to the corresponding author.

## Ethics Statement

All experiments were performed in accordance with the guidelines for the use of animal in research as stated by the Institutional Animal Care and Use Committee at the University of Georgia. The protocol was approved by the Institutional Animal Care and Use Committee at the University of Georgia.

## Author Contributions

WK and MS brought research ideas and designed the study. SS, YW, and MA conducted the experiment and generated the data. SS and CC wrote the manuscript. All authors read and approved the final version of the manuscript.

## Conflict of Interest

The authors declare that the research was conducted in the absence of any commercial or financial relationships that could be construed as a potential conflict of interest.
